# Cardiovascular complications of cellular immunotherapies and associated risk factors

**DOI:** 10.1038/s44325-025-00057-7

**Published:** 2025-06-10

**Authors:** Eli Grunblatt, Nausheen Akhter

**Affiliations:** 1 Department of Medicine, Feinberg School of Medicine, Northwestern University, 676 N. St. Clair, Suite 2330, Chicago, IL 60611 USA; 2 Division of Cardiovascular Medicine, Department of Medicine, Feinberg School of Medicine, Northwestern University, 676 N. St. Clair, Suite 600, Chicago, IL 60611 USA

**Keywords:** Cardiology, Cardiovascular diseases

## Abstract

In recent years, the use of cellular immunotherapies has become widespread for the treatment of patients with refractory malignancies. While this has led to improved overall outcomes, these therapies have been associated with numerous, sometimes severe cardiotoxicities. In this review, we highlight the spectrum of cardiovascular adverse events that can occur following cellular immunotherapies with a particular emphasis on the pre-treatment risk factors that may be associated with these cardiotoxicities.

## Introduction

**Fig. 1:**
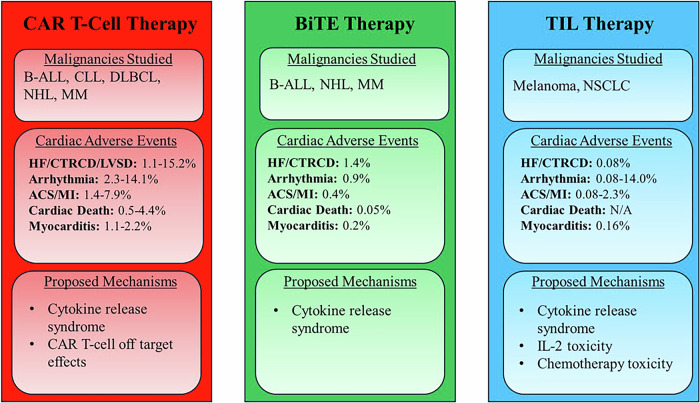
Schematic summarizing key data regarding cardiovascular adverse events associated with cellular immunotherapies. Adverse event types are reported as the range of incidence (in %) across studies. ACS acute coronary syndrome, B-ALL B-cell acute lymphocytic leukemia, BiTE bispecific T-cell engager, CAR chimeric antigen receptor, CLL chronic lymphocytic leukemia, CTRCD cancer therapy-related cardiac dysfunction, DLBCL diffuse large B-cell lymphoma, HF heart failure, IL interleukin, LVSD left ventricular systolic dysfunction, MI myocardial infarction, MM multiple myeloma, NHL non-Hodgkins lymphoma, NSCLC non-small cell lung cancer, TIL tumor-infiltrating lymphocyte.

Cellular immunotherapies are a novel class of cancer-directed therapy in which the patient’s own immune system is recruited and/or modified to target malignant cells. These include modalities such as chimeric antigen receptor (CAR) T-cell therapy, bispecific T-cell engager (BiTE) therapy, and tumor-infiltrating lymphocyte (TIL) therapy (Fig. 1)^1–3^. In recent years, the advent of these therapies has revolutionized the management of both hematologic and solid organ malignancies, leading to improved outcomes for patients with refractory and relapsed disease^4–7^. However, despite these successes, the use of these therapies is not without risk. Increasing evidence has shown that CAR T-cell, BiTE, and TIL therapies can be associated with the development of cardiovascular adverse events (CVAEs) ranging in severity from mild to severe and even fatal^8,9^. With the widespread uptake of cellular immunotherapies as second and third-line treatments for many malignancies, a comprehensive understanding of the types of CVAEs that can occur in these patients and the risk factors associated with adverse events are crucial to inform guidelines regarding pretreatment cardiovascular risk stratification and post-treatment cardiovascular surveillance in patients undergoing these therapies. In this review, we highlight the current data regarding CVAEs in cancer patients receiving cellular immunotherapies, with a particular focus on the risk factors that have been associated with the development of these adverse events.

## Cardiovascular adverse events associated with CAR T-cell therapy

CAR T-cell therapies are a class of adoptive cellular therapy in which a patient’s own T-cells are harvested, genetically engineered to express a modified T-cell receptor targeting a tumor-specific antigen such as cluster of differentiation (CD)-19 or B-cell maturation antigen (BCMA) and then re-infused back into the patient following a regimen of lymphodepleting chemotherapy^9,10^. Currently, there are multiple CD-19 targeting CAR T-cell products that have been FDA approved for use in lymphoid malignancies such as relapsed lymphomas and B-cell leukemias^5,11–13^. More recently, several BCMA targeting CAR T-cell products have been approved for use in myeloid malignancies such as relapsed multiple myeloma^10,14^. Additionally, there is active research ongoing for development of CAR T-cell products targeting solid organ malignancies, as well as other conditions including several autoimmune diseases and ischemic cardiomyopathy^15–17^. The advent of CAR T-cell therapy in recent years has revolutionized the management of hematologic malignancies, greatly improving survival and overall outcomes for patients with relapsed, refractory disease^5,10–12^. However, despite these successes, the use of CAR T-cell therapies has not come without unintended adverse effects. Many of the initial clinical trials involving CAR T-cell therapies reported CVAEs, including arrhythmia, heart failure/cardiomyopathy, coagulopathy, and cardiac arrest. The incidence of these CVAEs was highly variable across different trials, with rates of arrhythmia ranging from 4 to 38%^5,10,11,13,14,18–22^. Over the past several years, with the widespread uptake of CAR T-cell therapy as standard of care for refractory hematologic malignancies, there have been multiple dedicated retrospective observational studies as well as several prospective studies aimed at better characterizing the CVAEs that occur post-CAR T-cell therapy (Table 1).

**Table 1:** Cardiovascular adverse events following CAR T-cell therapy

Study	Population	Sample size	CVAE definition system	Heart failure/CTRCD/ LVSD, *N* (%)	Arrhythmia, *N* (%)	ACS/MI, *N* (%)	Cardiac Death, *N* (%)	Other CVAE, *N* (%)
Burstein, et al. 2018^24^	Pediatric B-ALL	98	Ad Hoc	9 (9.2%)	N/A	N/A	N/A	N/A
Alvi, et al. 2019^25^	DLBCL & MM	137	Ad Hoc	6 (4.4%)	5 (3.6%)	N/A	6 (4.4%)	N/A
Lefebvre, et al. 2020^26^	NHL, B-ALL, & CLL	145	ACC/AHA MACE	22 (15.2%)	11 (7.6%)	2 (1.4%)	2 (1.4%)	N/A
Shalabi, et al. 2020^27^	Pediatric B-ALL & NHL	52	Ad Hoc	6 (11.5%)	N/A	N/A	N/A	N/A
Ganatra, et al. 2020^28^	NHL	187	Ad Hoc	19 (10.2%)	13 (7.0%)	N/A	N/A	N/A
Brammer, et al. 2021^29^	NHL	90	CTCAE	1 (1.1%)	11 (12.2%)	N/A	N/A	Myocarditis: 2 (2.2%)
Wudhikarn, et al. 2020^31^	DLBCL	60	CTCAE	3 (5.0%)	8 (13.3%)	N/A	N/A	Pericardial Effusion: 1 (1.7%)
Qi, et al. 2021^30^	B-ALL, NHL, & MM	126	CTCAE	16 (12.7%)	8 (6.3%)	10 (7.9%)	N/A	N/A
Steiner, et al. 2022^35^	DLBCL	165	ACC/AHA MACE	4 (2.4%)	21 (12.7%)	3 (1.8%)	1 (0.6%)	CVA: 4 (2.4%)
Lee, et al. 2023^32^	MM	78	ESC/ICOS	4 (5.1%)	11 (14.1%)	N/A	1 (1.3%)	N/A
Lee, et al. 2023^33^	NHL	90	ESC/ICOS	1 (1.1%)	10 (11.1%)	N/A	N/A	Myocarditis: 1 (1.1%)
Mahmood, et al. 2023^34^	NHL & B-ALL	202	Ad Hoc	26 (12.9%)	N/A	11 (5.4%)	1 (0.5%)	N/A
Patel, et al. 2024^23^	NHL, CLL, & MM	75	Ad Hoc	3 (4.0%)	6 (8.0%)	N/A	1 (1.3%)	N/A
Lefebvre, et al. 2023^40^ (Prospective)	NHL & B-ALL	44	ACC/AHA MACE	1 (2.3%)	1 (2.3%)	N/A	N/A	N/A
Korell, et al. 2023^41^ (Prospective)	NHL, B-ALL, & MM	137	ACC/AHA MACE	17 (12.4%)	N/A	N/A	N/A	N/A

*ACS* acute coronary syndrome, *ACC/AHA* American College of Cardiology/American Heart Association, *B-ALL* B-cell acute lymphocytic leukemia, *CAR* chimeric antigen receptor, *CLL* chronic lymphocytic leukemia, *CTCAE* common terminology criteria for adverse events, *CTRCD* cancer therapy-related cardiac dysfunction, *CVA* cerebrovascular accident, *CVAE* cardiovascular adverse event, *DLBCL* diffuse large B-cell lymphoma, *ESC/ICOS* European Society of Cardiology/International Cardio-Oncology Society, *LVSD* left ventricular systolic dysfunction, *MACE* major adverse cardiac event, *MI* myocardial infarction, *MM* multiple myeloma, *NHL* non-Hodgkins lymphoma.

Across retrospective studies, the most commonly identified CVAEs associated with CAR T-cell therapy include arrhythmia (including supraventricular arrhythmias such as atrial fibrillation, as well as ventricular arrhythmias), left ventricular systolic dysfunction and/or congestive heart failure, myocardial infarction, and cardiac death (Table 1)^23–35^. Of note, there is a high degree of variability across studies regarding the incidence of post-CAR T-cell therapy CVAEs. For example, in a retrospective study of 145 patients with acute lymphocytic leukemia, chronic lymphocytic leukemia, or non-Hodgkin’s lymphoma, Lefebvre et al. reported a 21.4% incidence of major adverse cardiac events (MACE), including cardiac death, heart failure, ischemic stroke, and acute coronary disease^26^. In a similar retrospective study of 187 patients with lymphoma, Ganatra, et al. reported only a 10.3% incidence of CVAEs, namely cardiomyopathy^28^. In a recent meta-analysis of 13 studies totaling 1528 patients, Koeckerling, et al. found an overall relatively low prevalence of CVAEs across CAR T-cell patients including supraventricular arrhythmias (7.8%), left ventricular dysfunction (8.7%), heart failure (3.9%), and cardiac death (0.6%)^36^. In another meta-analysis of 23 studies, Maleki et al. found a much higher prevalence of CVAEs, including arrhythmia (54%) and heart failure (33%)^37^. These differences across retrospective studies (and consequently meta-analyses) can likely be attributed to differences in the number of patients included, the population being studied (i.e., lymphoma, leukemia, or myeloma patients), length of follow-up, and the methodology used to determine CVAEs. Regarding methodology, studies have used either the common terminology criteria for adverse events, the ACC/AHA MACE criteria, or else an ad hoc system for determining incidence and characterization of post-CAR T-cell therapy adverse events^23,26,28,32,33^. These systems contain different definitions of cardiotoxicities, thereby likely contributing to heterogeneity across studies, suggesting a need for the use of a standardized system for defining CVAEs in future studies^38,39^.

To date, there have been two notable prospective studies investigating the development of CVAEs following CAR T-cell therapies. Lefebvre et al. conducted a prospective study of 43 non-Hodgkins lymphoma patients and one chronic lymphocytic leukemia patient over the course of 1 year of follow-up including serial echocardiography. In this study, only two patients (4.5%) developed MACE (one patient developed heart failure and the other arrhythmia) during the follow-up period^40^. Similarly, Korell et al. conducted a prospective study of 137 patients with lymphoma and myeloma and found that while 5.4% of patients developed new arrhythmia post-CAR T-cell therapy, no patients developed MACE during the follow-up period^41^. Interestingly, the rate of post-CAR T-cell CVAE development was significantly lower in the 2 prospective studies to date compared with previous retrospective studies. In the case of the Lefebvre et al. study, this may be partially explained by the study’s relatively low enrollment. Furthermore, both prospective studies incorporated a relatively strict definition of MACE compared to prior studies, which may also partly explain the relatively low CVAE incidence. Overall, while significant discrepancies exist regarding the true incidence of CVAEs following CAR T-cell therapy, the occurrence of sometimes severe and fatal adverse events is of significant concern. Further comprehensive, large-scale prospective studies are needed to fully elucidate the dynamics of short and long-term CVAEs associated with CAR T-cell therapy.

### Mechanisms of CAR T-cell therapy-associated adverse events

Given that numerous studies have shown that CAR T-cell therapies can lead to varied, sometimes severe, CVAEs, understanding the mechanisms behind these adverse events is of paramount importance to the field. The precise mechanisms contributing to post-CAR T-cell cardiotoxicity remain largely unknown. A leading theory behind the development of post-CAR T-cell therapy CVAEs is focused on the effects of cytokine release syndrome (CRS)^42^. CRS is a phenomenon in which infusion of CAR T-cells and their subsequent activity triggers a widespread systemic inflammatory response mediated by the release of cytokines, including interleukin (IL)-6 among others^43^. CRS is very common in CAR T-cell patients, with some studies estimating an incidence of up to 90%^9,43^. Furthermore, CRS can manifest as a wide variety of symptoms, including fever, dyspnea, and hypotension with severity ranging from mild and asymptomatic (typically characterized as Grade 1) up to severe and possibly fatal (Grades 3–4)^44^. Currently, it is theorized that CRS contributes to cardiotoxicity both through the effects of systemic inflammatory stress and the direct effects of cytokines on the myocardium^42,45^. However, there remains a lack of both preclinical and clinical data on this topic, and the effect of CRS on the composition of the cardiac immune microenvironment remains unclear. Additional mechanisms proposed for post-CAR T-cell cardiotoxicity include complications resulting from unintended CAR T-cell targeting of a non-tumor antigen expressed in normal tissue. This scenario was first reported in a case series of two patients who were treated with CAR T-cells targeting the melanoma-associated antigen 3 (MAGE-A3) and subsequently developed myocarditis with evidence of engineered T cell infiltration into the myocardium^46^. While the results of this study were highly concerning and stress the importance of cautious epitope design in CAR T-cell engineering, to date, no cases of similar off-target effects have been reported in patients with hematologic malignancies undergoing CAR T-cell therapy. Further comprehensive studies are needed to better elucidate the precise mechanisms of CAR T-cell-associated cardiotoxicities.

### Risk factors for CAR T-cell therapy-associated adverse events

Understanding potential risk factors for CAR T-cell-associated CVAEs is crucially important, both to inform risk stratification of patients prior to treatment and surveillance of patients post-therapy. One of the most well-studied potential risk factors for the development of CVAEs is the presence of CRS, especially more severe, high-grade CRS. As discussed above, CRS is characterized by a systemic inflammatory response following CAR T-cell infusion and is thought to potentially contribute to cardiotoxicity^42,45^. In clinical practice, CRS is graded on a standardized scale of 0–4, with 0 representing no CRS and 4 representing the most severe forms of CRS^44^. Numerous retrospective studies have found a strong association between higher grade (≥2) CRS and downstream development of CVAEs post-CAR T-cell therapy^25,26,28,29,35^. These findings not only add weight to the theory that CRS may in part be driving cardiotoxicities in these patients, but also open an avenue for prevention of CVAEs via early treatment of CRS. Current guidelines from multiple societies of hematology recommend treatment with tocilizumab, an IL-6 inhibitor, for CRS grade 2 and above, with many centers also using this agent to treat CRS grade 1 in many circumstances, a practice that potentially should be further encouraged given the above findings^43,47^. However, it is important to note that the CRS grade as a risk factor for CVAE development has not been a universal finding across studies. In several retrospective studies, CRS grade was not significantly associated with downstream development of CVAEs following CAR T-cell therapy^23,31,32^. This heterogeneity is further highlighted by discrepancies in meta-analyses. In a meta-analysis by Koeckerling et al. the authors found that there was no significant association between CRS grade and CVAEs, while in a meta-analysis by Maleki et al. CRS grade was found to be significantly associated with downstream CVAEs^36,37^. In both of the prospective studies looking at CVAEs post-CAR T-cell therapy, there was no association between CRS and CVAE development, though this is likely in part due to the low event rate seen in these cohorts^40,41^. A possible explanation for this heterogeneity is the overall improvement of CRS management over time. In the earlier days of CAR T-cell therapy, CRS was less well recognized, characterized, and managed compared to current clinical practice^47^. As such, it is possible that the link between CRS grade and downstream CVAEs has evolved, and patients treated in more recent years have shorter exposure to CRS due to more rapid recognition of CRS by providers and early intervention with agents such as tocilizumab^43^. In fact, in Alvi et al. the authors found that while CRS grade was associated with downstream CVAEs, this association was weaker in patients who were aggressively managed with tocilizumab^25^. These changes in management over time may in part account for the heterogeneity between the aforementioned meta-analyses, as incorporation of newer studies may reflect this possible shift in the impact of CRS on downstream adverse events. Overall, further comprehensive prospective studies are needed to fully elucidate the relationship between CRS and the development of post-CAR T-cell therapy CVAEs.

Another potential class of risk factors for post-CAR T-cell cardiotoxicity concerns the more well-established general cardiovascular risk factors, including hypertension, diabetes mellitus, and tobacco use among others^48^. A summary of observed associations between cardiovascular risk factors and development of CVAEs following CAR T-cell therapy is presented in Table 2. Notably, older age, preexisting hypertension, and prior history of arrhythmia were significantly associated with downstream CVAE development in multiple retrospective studies^26,28,33–35^. Several isolated retrospective studies have also reported significant associations between post-CAR T-cell CVAEs and prior history of coronary artery disease, heart failure, and hyperlipidemia, respectively^28,34^. No studies have shown a significant association between cardiotoxicity and either diabetes mellitus or tobacco use in these patient populations (Table 2). While further data is needed to precisely define the relationship between preexisting cardiovascular conditions and CAR T-cell related CVAEs, the existing data, while heterogeneous, nonetheless potentially argues for aggressive pre-screening of patients and management of arrhythmias prior to undergoing these therapies.

**Table 2:** Baseline risk factors significantly associated with cardiotoxicities following CAR T-cell therapy

Study	Population	Sample size	CRS grade	CV risk factors	Biomarkers	Echocardiographic parameters
Burstein, et al.^24^	Pediatric B-ALL	98	Not Reported	None	Not Reported	Decreased LVEF
Alvi, et al.^25^	DLBCL & MM	137	Yes	None	Not Reported	None
Lefebvre, et al.^26^	NHL, B-ALL, & CLL	145	Yes	Prior arrhythmia	Not Reported	Increased LAVI, Increased MV E/e’
Shalabi, et al.^27^	Pediatric B-ALL & NHL	52	Yes	None	Not Reported	Decreased GLS
Ganatra, et al.^28^	NHL	187	Yes	Older age, Prior HTN, Prior HLD, Prior CAD	None	None
Brammer, et al.^29^	NHL	90	Yes	None	None	None
Wudhikarn, et al.^31^	DLBCL	60	No	None	None	None
Qi, et al. 2021^30^	B-ALL, NHL, & MM	126	Yes	None	Elevated creatinine	None
Steiner, et al.^35^	DLBCL	165	Yes	Older age	None	Diastolic dysfunction
Lee, et al.^32^	MM	78	Yes	None	None	None
Lee, et al.^33^	NHL	90	No	Older age	Elevated creatinine	Increased LAVI
Mahmood, et al.^34^	NHL & B-ALL	202	Yes	Prior HTN, Prior arrhythmia, Prior HF	Elevated troponin, Elevated BNP	Decreased LVEF
Patel, et al.^23^	NHL, CLL, & MM	75	No	None	None	Decreased GLS, Increased MV E/e’
Lefebvre, et al.^40^ (Prospective)	NHL & B-ALL	44	No	None	None	None
Korell, et al.^41^ (Prospective)	NHL, B-ALL, & MM	137	No	None	None	None

*B-ALL* B-cell acute lymphocytic leukemia, *BNP* brain natriuretic peptide, *CAD* coronary artery disease, *CAR* chimeric antigen receptor, *CLL* chronic lymphocytic leukemia, *CRS* cytokine release syndrome, *CV* cardiovascular, *DLBCL* diffuse large B-cell lymphoma, *GLS* global longitudinal strain, *HF* heart failure, *HLD* hyperlipidemia, *HTN* hypertension, *LAVI* left atrial volume index, *LVEF* left ventricular ejection fraction, *MM* multiple myeloma, *MV*mitral valve, *NHL* non-Hodgkins lymphoma.

Further cardiovascular risk stratification for CAR T-cell involves the use of cardiac and inflammatory biomarkers. In the field of cardio-oncology, the use of cardiac biomarkers including high-sensitivity troponin (hsTnI) and brain natriuretic peptide (BNP) has proven valuable for risk stratification of patients undergoing anthracycline-based cancer treatment^48^. Additionally, given the prevalence of CRS among CAR T-cell patients, there is a high degree of interest in the potential use of inflammatory biomarkers, including ferritin, C- reactive protein (CRP), and IL-6, among others for pretreatment risk stratification. In a retrospective study by Alvi et al. the authors found that elevated hsTnI in conjunction with CRS grade >2 was associated with increased likelihood of CVAE development^25^. In Mahmood et al. the authors found that elevated pretreatment levels of hsTnI and BNP were significantly associated with the development of post-CAR T-cell therapy CVAEs in a cohort of myeloma patients. In that same study, the authors found that although pretreatment levels of inflammatory biomarkers were not significantly elevated in patients who went on to develop CVAEs, those patients who developed CVAEs did experience a significant rise in levels of ferritin, CRP, and IL-6 post-treatment, suggesting that these biomarkers may be useful in determining surveillance strategies for this patient population (Table 2)^34^.

Echocardiographic parameters are also being investigated for cardiovascular risk stratification for post-CAR T-cell cardiotoxicity. As with cardiac biomarkers, the use of echocardiographic parameters including left ventricular ejection fraction (LVEF) and global longitudinal strain (GLS) has shown prognostic utility for anthracycline mediated cardiotoxicity^48^. Regarding the use of echocardiography for risk stratification of CAR T-cell patients, several retrospective studies have explored the associations between parameters such as LVEF, GLS, mitral valve E/e’, and left atrial volume index (LAVI) and downstream cardiotoxicity (Table 2)^23,24,26,27,33^. In studies by Burstein et al. and Mahmood et al. the authors found that lower pretreatment LVEF was significantly associated with post-CAR T-cell CVAEs^24,34^. In a study of pediatric patients by Shalabi et al. and a study of adult patients by Patel et al. the authors found that lower baseline GLS was significantly associated with the development of downstream cardiotoxicity^23,27^. Patel et al. and Lefebvre et al. also found that higher baseline mitral valve E/e’ was associated with increased likelihood of CVAE development post-CAR T-cell therapy^23,26^. Finally, Lefebvre et al. and Lee et al found that increased baseline LAVI was significantly associated with downstream CVAE development^26,33^. Importantly, as with other risk factors discussed earlier in this review, the association between pretreatment echocardiographic parameters and post-CAR T-cell cardiotoxicity is not consistent across all studies (Table 2). Given that many echocardiographic parameters are affected by variables such as loading conditions and known inter-operator variability in acquisition of certain parameters, further, comprehensive prospective studies are needed to better determine the utility of echocardiography and other imaging strategies (such as cardiac MRI) for risk stratification and surveillance of patients receiving CAR T-cell therapies.

Notably, CAR T-cell therapy is currently approved as a second- or third-line agent for refractory malignancies, and as such, many patients are exposed to potentially cardiotoxic agents as part of earlier treatment regimens prior to receiving CAR T-cell therapies^1^. For example, first-line treatment for many lymphoma patients includes anthracycline therapy, which is known to cause cardiac dysfunction^49^. Similarly, many myeloma patients are treated with carfilzomib, a proteasome inhibitor which is also known to cause potential cardiovascular adverse events^50^. As such, it is plausible that prior exposure to these cardiotoxic agents may be a significant risk factor for the development of CVAEs downstream of CAR T-cell therapy. To date, no studies have found a significant association between prior anthracycline exposure and post-CAR T-cell CVAEs; however, this is likely due in part to the fact that nearly all lymphoma patients receive anthracyclines prior to CAR T-cell therapy, leaving very few control non-anthracycline-exposed patients available for comparison. Depending on the timing of disease relapse, some patients may receive CAR T-cell therapies relatively soon after completing their initial first-line treatment^51^. As such, it can be difficult to determine the degree to which certain adverse events are a result of CAR T-cell therapy or else a delayed manifestation of cardiotoxicity from other agents such as anthracyclines.

### Management of CAR T-cell therapy-related adverse events

Current guidelines from the European Society of Cardiology (ESC) in collaboration with the International Cardio-Oncology Society (ICOS) recommend that patients planned to receive CAR T-cell therapies undergo a baseline assessment of cardiac function with a thorough cardiovascular history, baseline cardiac biomarkers including hsTnI and BNP or equivalent, and echocardiography^42,48^. During treatment, aggressive management of CRS, for example, using tocilizumab, is recommended for potential prevention of cardiovascular complications as described above. At present, there are limited guidelines regarding post-infusion follow-up surveillance of patients receiving CAR T-cell therapies. While patients who develop higher grade CRS are recommended to undergo routine assessment with cardiac biomarkers, the optimal timeline of surveillance for these patients remains unclear^42,48^. Furthermore, active research is ongoing regarding the utility of echocardiography for short- and long-term surveillance of CAR T-cell patients, and further studies are needed on this topic.

## Cardiovascular adverse events associated with BiTE therapy

BiTE therapy is a form of T-cell activating immunotherapy consisting of an antibody engineered to contain antigen binding sites that target both a tumor-specific antigen as well as a native T-cell specific antigen, which results in co-localization of T-cells and tumor cells, leading to increased tumor destruction^9^. Currently, BiTE therapies are approved and available for B-cell leukemia (blinatumomab), lymphoma (glofitamab, epcoritamab, and mosunetuzumab), and multiple myeloma (teclistamab, talquetamab, and elranatamab). As with CAR T-cell therapy, the advent of BiTE therapy has shown remarkable promise for patients with refractory hematologic malignancies^6,52–57^. Notable differences between BiTE therapy and CAR T-cell therapy include the fact that while CAR T-cells are a unique product derived from a patient’s own T-cells, BiTE therapies are not custom-made for each patient. Furthermore, unlike CAR T-cell therapy, administration of BiTE therapy does not require pretreatment with lymphodepleting chemotherapy and BiTE therapy can be given repeatedly in multiple cycles unlike CAR T-cell therapy which is typically only given once in a single infusion^1,9^. As with CAR T-cell therapy, CRS has been observed with BiTE therapy due to its effect on activating T-cells and adjacent immune effectors. Of note, CRS occurs at a significantly lower rate in BiTE patients compared to CAR T-cell patients^58^.

Most of the data regarding CVAEs following BiTE therapy comes from the original clinical trials of these therapies, which reported isolated incidents of cardiac arrest and heart failure for blinatumomab and a low incidence of arrhythmias for teclistamab and epcoritamab, respectively^52,54,55^. Currently, there are a limited number of dedicated studies which investigate the development of CVAEs following BiTE therapy (Table 3). In a retrospective study of 50 patients with leukemia treated with blinatumomab by Jung et al. the authors reported a 14% incidence rate of CVAEs, including arrhythmia and hypotension^59^. Furthermore, in a pharmacovigilance study of 3668 events reported in the FAERS system for patients treated with BiTE therapies, 20.4% of events were classified as CVAEs, including heart failure and atrial fibrillation. In this same study, the authors reported that teclistamab was more associated with the risk of myocarditis and shock, while blinatumomab was more associated with the risk of coagulopathy^58^. Overall, there remains a significant paucity of data regarding post-BiTE therapy cardiotoxicities, and further studies are needed to better characterize the potential CVAEs associated with these treatment modalities.

**Table 3:** Cardiovascular adverse events following BiTE therapy

Study	Population	Sample size	BiTE product	Heart failure (%)	Arrhythmia, *N* (%)	ACS/MI, *N* (%)	Myocarditis *N*(%)	Cardiac death *N*(%)	Other *N*(%)
Jung, et al.^56^	B-ALL	50	Blinatumomab	N/A	N/A	N/A	N/A	N/A	Unspecified CVAE: 7 (14.0%)
Sayed, et al. 2024^55^	B-ALL, NHL, & MM	3668	Blinatumomab, Teclistamab, Glofitamab, Mosunetuzumab, Epocritamab	52 (1.4%)	32 (0.9%)	16 (0.4%)	8 (0.2%)	2 (0.05%)	N/A

*ACS* acute coronary syndrome, *B-ALL* B-cell acute lymphocytic leukemia, *BiTE* bispecific T-cell engager, *CVAE* cardiovascular adverse event, *MI* myocardial infarction, *MM* multiple myeloma, *NHL* non-Hodgkins lymphoma.

### Risk factors for BiTE therapy-associated adverse events and possible mechanisms

Given the limited number of studies currently available regarding CVAEs following BiTE therapies, there is limited data regarding the risk factors which may predispose patients to BiTE therapy-related cardiotoxicities. In both retrospective and pharmacovigilance studies, there were no significant risk factors identified for the development of post-BiTE therapy CVAEs^58,59^. Notably, CRS was not associated with CVAEs in these studies, likely in part due to the low incidence (as low as 4%) of CRS observed^58,59^. Generally speaking, the incidence of CRS is significantly lower following BiTE therapy compared to CAR T-cell therapy. Whereas rates of CRS after CAR T-cell therapy are commonly as high as 90%, rates of CRS after BiTE therapy are more variable, with incidence rates ranging from as low as 3% to as high as 72% depending on the product being used^6,47,52–55,58,59^. While not as consistent as in CAR T-cell therapy, cases of severe CRS involving hemodynamic compromise have been well documented following BiTE therapies^9,55,58^. As such, CRS may represent a potential driving mechanism behind some CVAEs in certain BiTE therapies, and further studies are needed to better elucidate this relationship and other potential mechanisms of BiTE therapy-related cardiotoxicity.

### Management of BiTE therapy-related adverse events

Currently, there are no definitive guidelines regarding cardiovascular pretreatment evaluation or post-treatment management of patients undergoing BiTE therapies, largely due to the limited number of studies available. At large cardio-oncology centers, clinical practice often involves standard pretreatment cardiovascular assessment with cardiovascular history, echocardiography, and cardiac biomarkers drawing from prior experience with other forms of cancer therapy-related cardiotoxicity^42^. Further studies are needed to better define approaches for risk stratification and post-treatment surveillance in these patient populations.

## Cardiovascular adverse events associated with TIL therapy

Tumor-infiltrating lymphocyte therapy is a novel form of adoptive cellular therapy in which T-cells are isolated from a patient’s tumor sample, expanded ex vivo, and then infused back into the patient following a pretreatment regimen of lymphodepleting chemotherapy. After infusion, patients are further treated with high-dose IL-2 to induce T-cell activation and invasion into the tumor, leading to targeted tumor cell destruction^3,7^. Currently, TIL therapy is approved for use in refractory melanoma and non-small cell lung cancer (NSCLC), with ongoing clinical trials investigating the use of these therapies in other solid organ malignancies^7,60,61^. TIL therapy remains the only adoptive cellular therapy available for non-hematologic malignancies and has shown great promise in improving outcomes for solid tumor patients. As with other forms of cellular immunotherapy, there has been a lot of interest in understanding potential cardiotoxicities that may be associated with TIL therapy (Table 4). In a retrospective study of 43 melanoma patients treated with TIL therapy, the authors reported a 14.0% incidence of atrial fibrillation, a 2.3% incidence of elevated troponin, and a 32.6% incidence of hypotension post-treatment^62^. In another recent retrospective study of 120 patients treated with TIL therapy (including 108 melanoma patients and 12 NSCLC patients), the authors reported a 5.8% incidence of post-treatment CVAEs, including two cases of myocarditis, one case of heart failure, one case of myocardial infarction, and one case of atrial fibrillation^63^. Overall, while there are only a handful of studies investigating CVAEs in TIL therapy patients, early indications show that these regimens are potentially associated with cardiotoxicities. Importantly, all current TIL regimens involve the administration of lymphodepleting chemotherapy and high-dose IL-2^3^. High-dose IL-2 has been reported to cause potential cardiotoxicities (including myocarditis) when used alone in other clinical settings^64–66^. As such, the degree to which potential CVAEs following TIL therapy are related to TIL themselves as opposed to sequelae of high-dose IL-2 treatment remains unclear, and further studies are needed to better characterize the adverse events that occur following TIL regimens.

**Table 4:** Cardiovascular adverse events following TIL therapy

Study	Population	Sample size	Heart failure (%)	Arrhythmia, *N* (%)	ACS/MI, *N* (%)	Myocarditis *N*(%)	Cardiac death *N*(%)
Fradley, et al.^59^	Melanoma	43	N/A	6 (14.0%)	1 (2.3%)	N/A	N/A
Borgers, et al.^60^	Melanoma & NSCLC	120	1 (0.08%)	1 (0.08%)	1 (0.08%)	2 (0.16%)	N/A

*ACS* acute coronary syndrome, *MI* myocardial infarction, *NSCLC* non-small cell lung cancer, *TIL* tumor-infiltrating lymphocyte.

### Risk factors for TIL therapy-associated adverse events and possible mechanisms

Given the early signals that TIL therapy may lead to CVAEs in select patients, understanding the risk factors associated with these events is of paramount importance. To date, neither of the retrospective studies investigating CVAEs in this patient population reported any significant associations between baseline patient characteristics and downstream cardiotoxicity, though in Fradley, et al. the authors noted that patients who went on to develop CVAEs had a higher prevalence of baseline hypertension and hyperlipidemia, though these associations did not meet statistical significance cutoffs^62,63^. Furthermore, it remains unclear if CRS plays a role in the development of potential cardiotoxicity following TIL therapy, as the incidence of CRS is markedly lower following TIL therapy compared to other forms of cellular immunotherapy^3,7,60,62,63^. As such, the theoretical mechanisms driving TIL associated CVAEs are largely unknown.

### Management of TIL therapy-related adverse events

Given the limited number of studies directly investigating CVAEs following TIL therapy, there are currently no firm guidelines regarding cardiovascular risk stratification and surveillance of these patients^42^. Although no risk factors have been definitively linked to TIL cardiotoxicity, the observations by Fradley et al. suggest that at minimum, a standard cardiovascular risk assessment should be performed in patients planned to undergo these therapies^62^. As the use of TIL therapy continues to be adopted across an increasing number of centers, further studies will be needed to determine appropriate risk stratification and post-treatment surveillance strategies for these patients.

## Other cellular immunotherapies

While CAR T-cell, BiTE, and TIL therapies are currently the mainstays of clinically used cellular immunotherapies, additional modalities are currently in development as potential promising treatment options for refractory malignancies. For example, T-cell receptor (TCR) T-cell therapy involves engineering T-cells with a highly sensitive customized T-cell receptor targeting solid tumor antigens^67^. Dendritic cell therapy involves ex vivo editing and/or expansion of dendritic cells for use in promoting an anti-tumor response by the immune system^68^. Natural killer cell therapy involves ex vivo expansion and adoptive transfer of natural killer cells to provoke a systemic inflammatory response, leading to a heightened immune response against tumor cells^69^. Lastly, similar to CAR T-cell therapy, CAR-M therapy involves isolating and genetically engineering a patient’s macrophages to target tumor cells^70^. To date, these newer forms of cellular immunotherapy have yet to be approved for clinical use, and as such, the potential cardiotoxicities of these treatments remain unknown. While each new modality of immunotherapy will undoubtedly have its own unique adverse events profile, the lessons learned from CAR T-cell, BiTE, and TIL therapies indicate that surveillance for cardiotoxicities will likely be important as these newer therapies enter clinical usage.

## Conclusion

Overall, while cellular immunotherapies have shown great promise for the treatment of refractory malignancies, the development of cardiovascular adverse events following treatment with CAR T-cell, BiTE, or TIL therapies remains a significant concern. For CAR T-cell therapy, although significant variability exists across studies regarding the types of cardiotoxicities that occur post-treatment and the pretreatment risk factors associated with them, there is nonetheless compelling evidence that post-treatment cardiotoxicity can be severe and requires careful monitoring. Furthermore, the significant variability across studies suggests that a standardized system should be adopted for categorizing CVAEs in future studies, especially as these therapies are expanded to solid tumors. Although there is currently relatively limited data regarding cardiotoxicities following BiTE and TIL therapies, early evidence suggests that these therapies are likely also associated with potentially serious cardiotoxicities. Further, comprehensive prospective studies are needed to fully elucidate the spectrum of cardiovascular events following treatment with cellular immunotherapies and the pretreatment risk factors that are associated with them. Further studies are also needed to understand the mechanisms by which these treatment modalities can lead to the development of cardiotoxicities in cancer patients.

## Data Availability

No datasets were generated or analysed during the current study.
